# A network pharmacology-based approach to explore the therapeutic potential of *Sceletium tortuosum* in the treatment of neurodegenerative disorders

**DOI:** 10.1371/journal.pone.0273583

**Published:** 2022-08-25

**Authors:** Yangwen Luo, Luchen Shan, Lipeng Xu, Srinivas Patnala, Isadore Kanfer, Jiahao Li, Pei Yu, Xu Jun

**Affiliations:** 1 College of Pharmacy, Jinan University, Guangzhou, China; 2 Faculty of Pharmacy, Rhodes University, Grahamstown, South Africa; University of Nebraska Medical Center, UNITED STATES

## Abstract

*Sceletium tortuosum* (SCT) has been utilized medicinally by indigenous Koi-San people purportedly for mood elevation. SCT extracts are reported to be neuroprotective and have efficacy in improving cognition. However, it is still unclear which of the pharmacological mechanisms of SCT contribute to the therapeutic potential for neurodegenerative disorders. Hence, this study investigated two aspects–firstly, the abilities of neuroprotective sub-fractions from SCT on scavenging radicals, inhibiting some usual targets relevant to Alzheimer’s disease (AD) or Parkinson’s disease (PD), and secondly utilizing the network pharmacology related methods to search probable mechanisms using Surflex-Dock program to show the key targets and corresponding SCT constituents. The results indicated sub-fractions from SCT could scavenge 2,2-diphenyl-1-picrylhydrazyl (DPPH) radical, inhibit acetylcholinesterase (AChE), monoamine oxidase type B (MAO-B) and N-methyl-D-aspartic acid receptor (NMDAR). Furthermore, the results of gene ontology and docking analyses indicated the key targets involved in the probable treatment of AD or PD might be AChE, MAO-B, NMDAR subunit2B (GluN2B-NMDAR), adenosine A_2A_ receptor and cannabinoid receptor 2, and the corresponding constituents in *Sceletium tortuosum* might be N-trans-feruloyl-3-methyldopamine, dihydrojoubertiamine and other mesembrine type alkaloids. In summary, this study has provided new evidence for the therapeutic potential of SCT in the treatment of AD or PD, as well as the key targets and notable constituents in SCT. Therefore, we propose SCT could be a natural chemical resource for lead compounds in the treatment of neurodegenerative disorders.

## Introduction

*Sceletium tortuosum (L*.*) N*.*E*. *Br* (SCT), a South African herb, with a long history of use by Koi-San natives, is reported to have various pharmacological activities such as anti-depressant [1], anti-anxiety [2], anti-epileptic [3] and analgesic [4] activities. Its extracts are reported to have shown efficacy in improving cognition [5, 6]. Cognition deficit is a predominantly general symptom of Alzheimer’s disease (AD) and in some cases of Parkinson’s diseases (PD)–hence it is postulated that neurodegenerative disorders that could be treated by compounds that possess neuroprotective effects [7–12]. Considering that there are certain neuroprotective constituents in SCT, which are reported to have the therapeutic potential in the treatment of neurodegenerative diseases, there is a need to invesigate the probable mechanisms that contribute to the possible treatment of neurodegenerative disorders, especially in AD or PD with cognitive impairments.

“Network pharmacology” (NP) methods have been usually applied in this form of research to access primary mechanisms of certain traditional Chinese medicines formula according to their traditional indications [13–16]. Some studies have also used NP to explore the possible novel indications for complicated Chinese traditional medicines [17, 18]. Application of NP could be further understood based on the published report by Fang JS et al who proposed and deciphered mechanism of action for some of the most widely studied medicinal herbs used in the treatment of AD [19].

**Fig 1:**
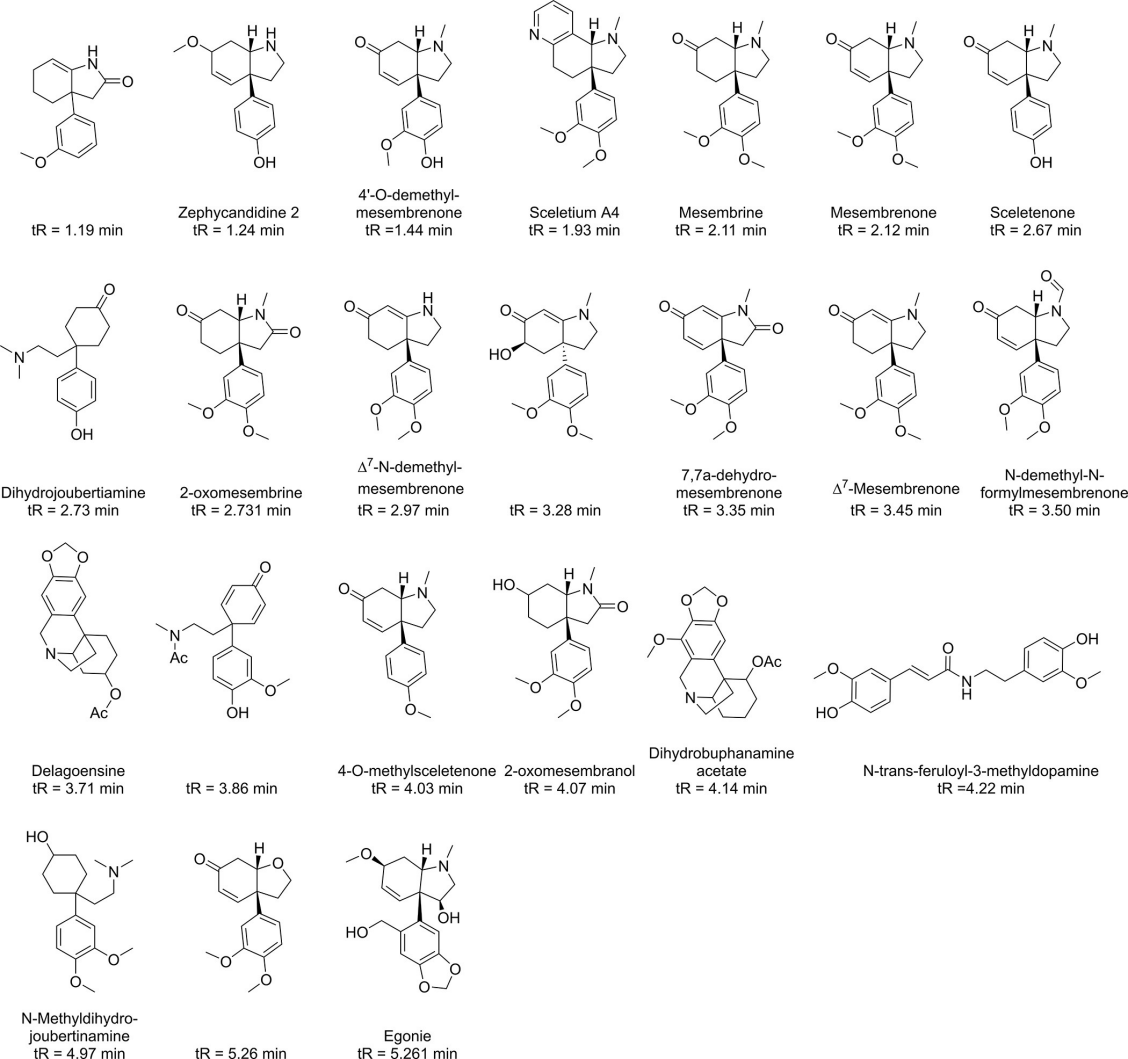
The constituents of neuroprotective sub-fractions from SCT in previous study. (tR represents their retention time in UPLC).

Our previous study [20] has showed the neuroprotective sub-fractions and possible neuroprotective constituents (Fig 1) in the neuroprotective sub-fractions extracted from SCT. The petroleum ether and ethyl acetate fractions were confirmed to possess neuroprotective efficacy, been further separated by silica gel column to obtain sub-fractions tested by cell experiments. Furthermore, natural products generally consist of various and diverse active constituents depending on the extraction process [21], which can lead to neuroprotective fractions that exert neuroprotective effect of SCT and probably caused due to multiple constituents. Thus, it makes such investigations laborious and difficult to decipher the elicited mechanisms. Hence, investigating the possibility of SCT extract for treating neurodegenerative disorders, as an integrated system as applied by traditional Chinese medicine, would provide insight by utilizing NP methods is a logical and scientific approach.

In this study, spectrophotometric assays were performed on SCT sub-fractions to assess neuroprotective action related efficacies based on the scavenging radicals, inhibiting acetylcholinesterase (AChE), monoamine oxidases (MAOs) and N-methyl-D-aspartic acid receptor (NMDAR). Subsequently, relevant NP methods and molecular docking were performed to understand the possible mechanisms that provide evidence to corelate the therapeutic potential of SCT in the treatment of AD or PD.

Furthermore, it is important to identify the key targets, and the corresponding constituents in neuroprotective sub-fractions involved in the probable treatment of AD or PD [2, 4, 22–33].

## Methods

### The neuroprotective sub-fractions from SCT and their identified constituents

Based on our previous study [20], SCT plant powder was extracted with alcohol and which was further extracted with petroleum ether and ethyl acetate. The petroleum ether and ethyl acetate fractions were confirmed to possess neuroprotective efficacy on MPP^+^-injured N2a cells or L-glutamate-injured PC12 cells. The active fractions were further separated by silica gel column to obtain sub-fractions. The sub-fractions were also tested by cell experiments [34–36] to give four neuroprotective sub-fractions–P5, P6, E1 and E3 (“P” and “E” mean the sub-fractions of petroleum ether and ethyl acetate fractions respectively). The active sub-fractions were once again preliminarily identified the constituents that were separated and identified from SCT in the current study. The chemical structures of these constituents are depicted in Fig 1.

### DPPH scavenging assay

The ability of the neuroprotective sub-fractions from SCT to scavenge 2,2-diphenyl-1-picrylhydrazyl (DPPH) radical was tested in 96-well polystyrene microtiter plates (Corning^®^). The extraction and separation methods to obtain the neuroprotective sub-fractions were performed as described in the previous study [20]. DPPH (TCI, Japan) was dissolved in methanol to obtain a concentration of 100 μM. The wells contained 100 μL DPPH and then added 100 μL of sub-fraction samples in different concentrations. Blank wells contained methanol in place of DPPH and control wells contained only methanol in place of samples. After shocking on a microoscillator, the plate was kept in the dark for 50 minutes. The absorbance was detected at a wavelength of 517 nm using a microplate reader (Bio-Tek Instruments Inc, USA). The clearance percent of DPPH was expressed as mean ± SEM calculated by following formula:

$$\mathrm{Clearance}\;(\%)=\frac{A_{\mathrm{control}}-(A_{\mathrm{sample}}-A_{\mathrm{blank}})}{A_{\mathrm{control}}}\times 100\%$$

### AChE inhibition assay

The experiment to test the AChE inhibiting ability of neuroprotective sub-fractions of SCT was performed as per procedure described by Ellman [37, 38]. 160 μL of PBS (0.1 M pH = 8), 10 μL of sample and 10 μL of AChE (0.5 U/mL, Solarbio, Beijing) were mixed in 96 wells plate for 20 min at 4°C, and then the wells were added 10 μL of 2,2’-dithiodibenzoic acid (10 mM, MedChemExpress) and 10 μL of acetylthiocholine iodide (10 mM, Solarbio, Beijing) for another 30 min at 37°C. The absorbance was detected at a wavelength of 405 nm. Blank wells had PBS added in place of AChE and control wells had PBS added in place of samples.

### MAOs inhibition assay

The MAOs inhibition percent of neuroprotective sub-fractions from SCT was measured by following procedures described by Holt with some modifications [39].

This study is got pass by Jinan University Laboratory Animal Ethical Committee.The IACUC issue number is 20220225–03. All studies related to animals were performed in accordance with the standards set forth in the eighth edition of Guide for the Care and Use of Laboratory animals, published by the National Academy of Sciences, the National Academies Press, Washington D.C (License number: SCXK(粤)2018-0002).We use Pentobarbital Sodium as anesthesia and reduce the pain of death in rats by excessive anaesthesia.

Female Sprague—Dawley rat (286 g) was killed by anesthetic, and its livers were dissected out, washed in ice-cold PBS (0.2 M, pH 7.6). Liver tissue (7 g) was homogenized 1:20 (w/v) in 0.3 M sucrose with a mechanical homogenizer. Following centrifugation at 1100*g* for 12 min, the supernatant was further centrifuged at 10 000*g* for 30 min to obtain a crude mitochondrial pellet, which was resuspended in 40 ml of PBS used as the source of MAOs.

40 μL of MAOs and 40 μL of samples were added in the wells for 20 min at 37°C and then the supplement of the enzyme substrate and chromogenic reagent were added for 60 min at 37°C. The enzyme substrate was tyramine (5 mM, Aladdin, Shanghai) and the chromogenic reagent was a mixture contained vanillic acid (5 mM, Shanghaiyuanye, China), 4-aminoantipyrine (1mM, Shanghaiyuanye, China), peroxidase (5 U/mL, Solarbio, Beijing) in PBS. The absorbance was detected at a wavelength of 490 nm. Blank wells had PBS added in place of tyramine and control wells had PBS added in place of samples.

The inhibition percentages of AChE and MAOs were expressed as mean ± SEM calculated by following formula:

$$\mathrm{Inhibition}\;\mathrm{percent}\;(\%)=\frac{A_{\mathrm{control}}-(A_{\mathrm{sample}}-A_{\mathrm{blank}})}{A_{\mathrm{control}}}\times 100\%$$

### Primary culture of rat hippocampal neurons

The hippocampus tissue was separated from Sprague-Dawley neonatal rat and placed in cold phosphate buffer saline under an asepsis condition, and then was digested with 0.25% trypsin for 20 min at 37°C. After trypsinization, hippocampal neurons were suspended in DMEM (Gibco) containing 10% horse serum (Gibco) and cultured in polyethylenimine-coated coverslips at a density of 105/cm^2^ for 4 h at 37°C. The medium was replaced with neurobasal medium (Gibco) containing B-27 supplement (Gibco) and L-glutamine (Gibco), and the cells were cultured at 37°C in a humidified environment of 95% air and 5% CO_2_ for 7 days.

### Whole cell patch clamp

To investigate the effect of two sub-fractions from SCT on the NMDAR mediated current, whole cell patch clamp was used to the record of NMDAR current by an amplifier (EPC-10, HEKA, Germany). Before recording, a negative pressure was exerted on the hippocampal neuron’s surface through microelectrode and formed a GΩ seal resistance, then the membrane potential was kept in -70 mV. The hippocampal neurons were exposed to NMDA (100 μM), Glycine (10 μM) and samples in different concentrations or D-2-Amino-5-phosphonovaleric acid (D-AP5) (100 μM).

NMDA (100 μM) and Glycine (10 μM) were used to activate the NMDA current. D-AP5, a NMDAR inhibitor, was used as a positive control. The current signals were recorded by the amplifier under a Gap-free mode and stored in PatchMaster software (HEKA, Germany).

Recording was allowed to start at least 5 min after the rupture of patch membrane to ensure stabilization of the intracellular milieu. Neurons that showed unstable or large (more than ∼50 pA) holding currents were rejected. The series resistance (<15 MΩ) and membrane capacitance were compensated and checked regularly during the recording.

The inhibition percentage of NMDAR was calculated according to the formula: (1-(I_NMDA + Glycine +Compound_ / I_NMDA +Glycine_)) x 100%. Data were expressed as mean ± S.E.M.

Extracellular fluid (pH 7.4) contained 140 mM NaCl, 4 mM KCl, 2 mM CaCl_2_•2H_2_O, 10 mM HEPES, 5 mM D-Glucose, 0.5 μM TTX, 10 μM NBQX, 10 μM Strychnine and 10 μM Bicuculline. Intracellular fluid (pH 7.2) contained 10 mM NaCl, 110 mM CsMeS, 2 mM MgCl_2_•6H_2_O, 10 mM HEPES, 10 mM EGTA, 2 mM Na_2_-ATP, 0.2 mM Na_2_-GTP.

### Network pharmacology methods to decipher possible mechanisms of SCT

Targets of the constituents identified from SCT in our previous study were obtained from Polypharmacology Browser 2 (https://ppb2.gdb.tools/) [40]. Methods: ECfp4 Naive Bayes Machine Learning model produced on the fly with 2000 nearest neighbors from extended connectivity fingerprint ECfp4. Targets of neurodegenerative disorder were elements of the intersection set obtained from GeneCards [41] (https://www.genecards.org/, Relevance score ≥ 10) and DisGeNET [42] (https://www.disgenet.org/, Score gda ≥ 0.1) databases using following keywords: Alzheimer’s disease, Parkinson’s disease, amyotrophic lateral sclerosis, spinocerebellar ataxia, Lewy bodies, frontotemporal dementia, Huntington’s disease and epilepsy.

Protein–protein interaction data were acquired from STRING 11.0 [43] (https://string-db.org/cgi/input.pl) with the species limited to “Homo sapiens”.

GO and KEGG pathway enrichment analyses were performed by DAVID Bioinformatics Resources 6.8 [44] (https://david.ncifcrf.gov/). The targets from the intersection set of targets of the constituents and diseases were submitted to obtain the terms of biological process, molecular function, cellular component and Kyoto Encyclopedia of Genes and Genomes (KEGG) pathways.

All visualized network models were established via Cytoscape 3.8.0. The topological feature of each node in network model was assessed by calculating three parameters named “Degree”, “Betweenness Centrality (BC)” and “Closeness Centrality (CC) by Network Analyze tool in Cytoscape software.

### Preliminary verification for the possible mechanisms by surflex-dock

The constituents were prepared by Sybyl-X 2.0. As docking ligands, their energy was minimized according following parameter settings: Powell method, 0.005 kcal/mol·A gradient termination, 1000 max iteration and Gasteiger-Huckel charges. Other settings were kept default.

The protein structures were obtained from PDB Protein Data Bank (http://www.rcsb.org/). To make docking pockets, the protein structures were extracted ligand substructure, repaired sidechains, added hydrogens and minimized their energy. Protomol generation mode was selected as “Ligand” and other settings were default. Reference molecules were set as their original ligands. Results of Total Score were output as the criterion to comparing the predictive affinities.

### Statistical method

Each value was an average of data from 3 independent experiments and each experiment included 3 replicates. Data were expressed as mean ± SEM and analyzed using GraphPad Prism V8.0 (GraphPad Software, Inc., San Diego, CA, USA). One-way analysis of variance (ANOVA) and Dunnett’s test were used to evaluate statistical differences.

## Results

### SCT sub-fractions scavenge DPPH radical

**Fig 2:**
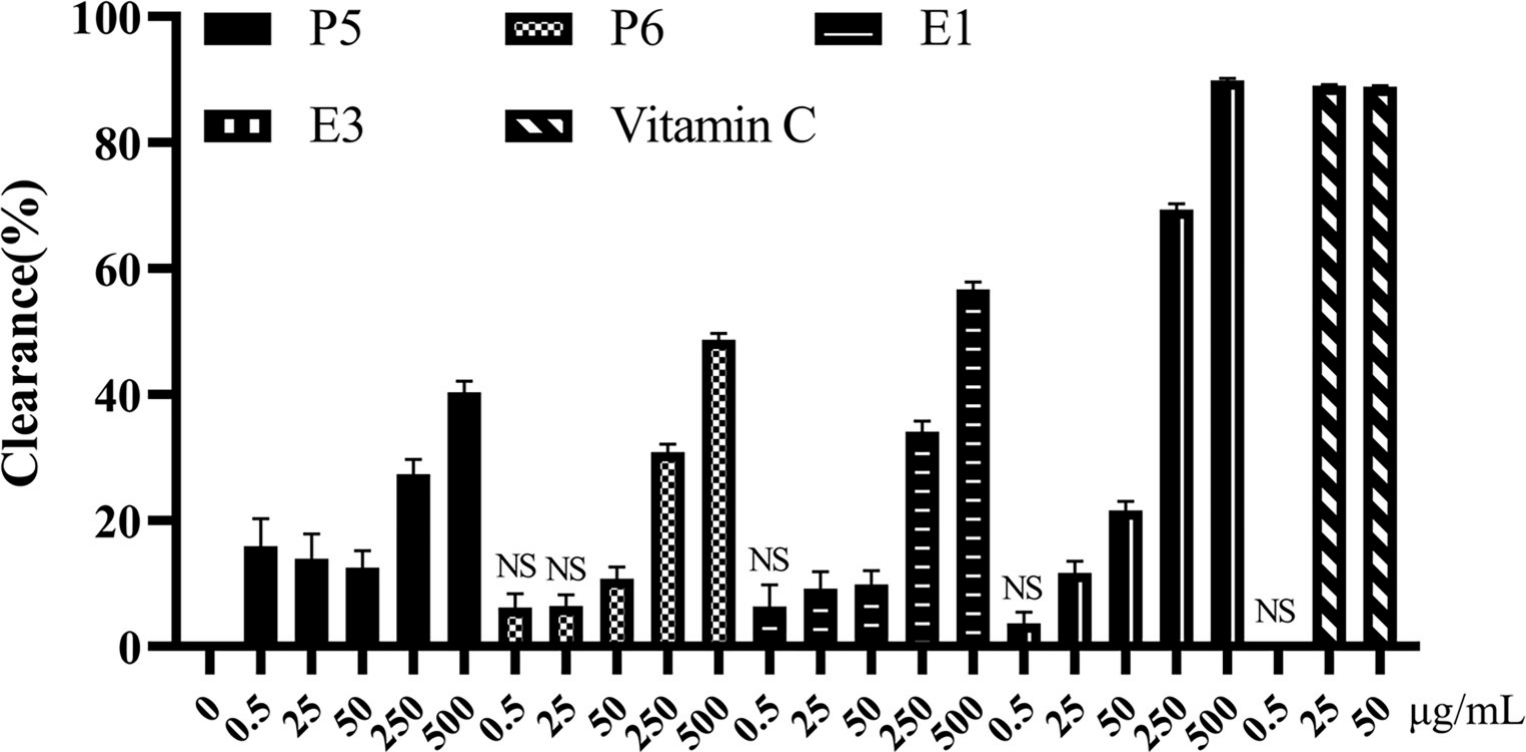
The DPPH clearance percentages of active sub-fractions. Data were expressed as mean ± S.E.M. from the data obtained from three independent experiments (n = 3). NS represents the mean of group has no significant different with the mean of control group.

The scavenging ability of DPPH radical of the SCT sub-fractions is depicted in Fig 2. The clearance percentages of four sub-fractions could all reach more than 40% at their highest concentration (500 μg/mL). Fraction E3 was the most potent sub-fraction on scavenging DPPH radical among these four neuroprotective sub-fractions from SCT, although weaker than the positive compounds–vitamin C.

### SCT sub-fractions inhibit AChE

**Fig 3:**
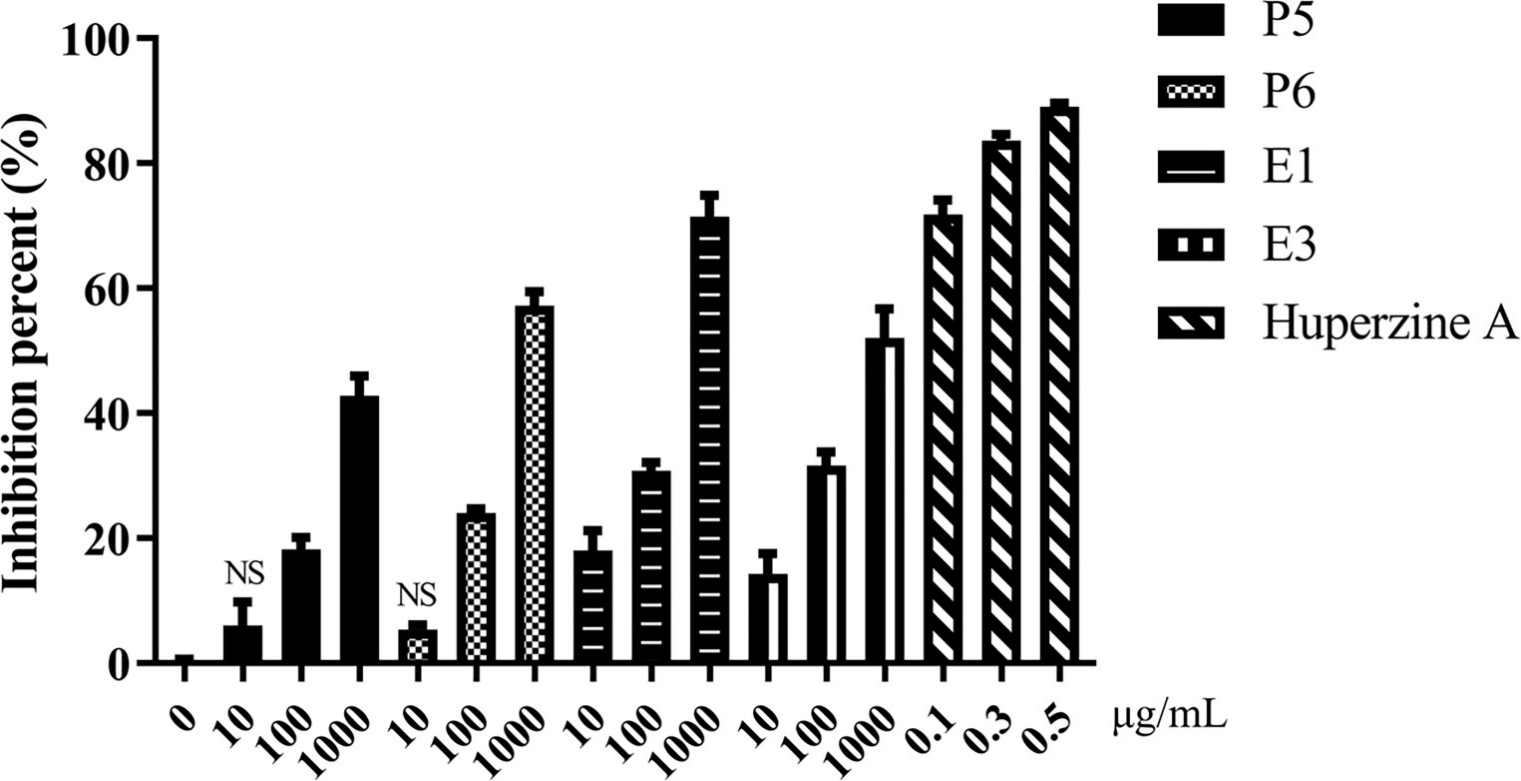
The inhibition percentages of active sub-fractions on AChE. Data were expressed as mean ± S.E.M. obtained from three independent experiments (n = 3). NS represents the mean of group has no significant difference compared to the mean of control group.

The AChE inhibition percent of four sub-fractions could reach more than 40% at their highest concentration (1000 μg/mL). Since contrast to Huperzine–a AChE inhibitor, their effects on AChE were considered as slight efficacy. It also showed that fraction E1 exhibited more than 60% inhibition percent on AChE, which was the most potent sub-fraction among the extracts (Fig 3).

### SCT sub-fractions inhibit MAOs

**Fig 4:**
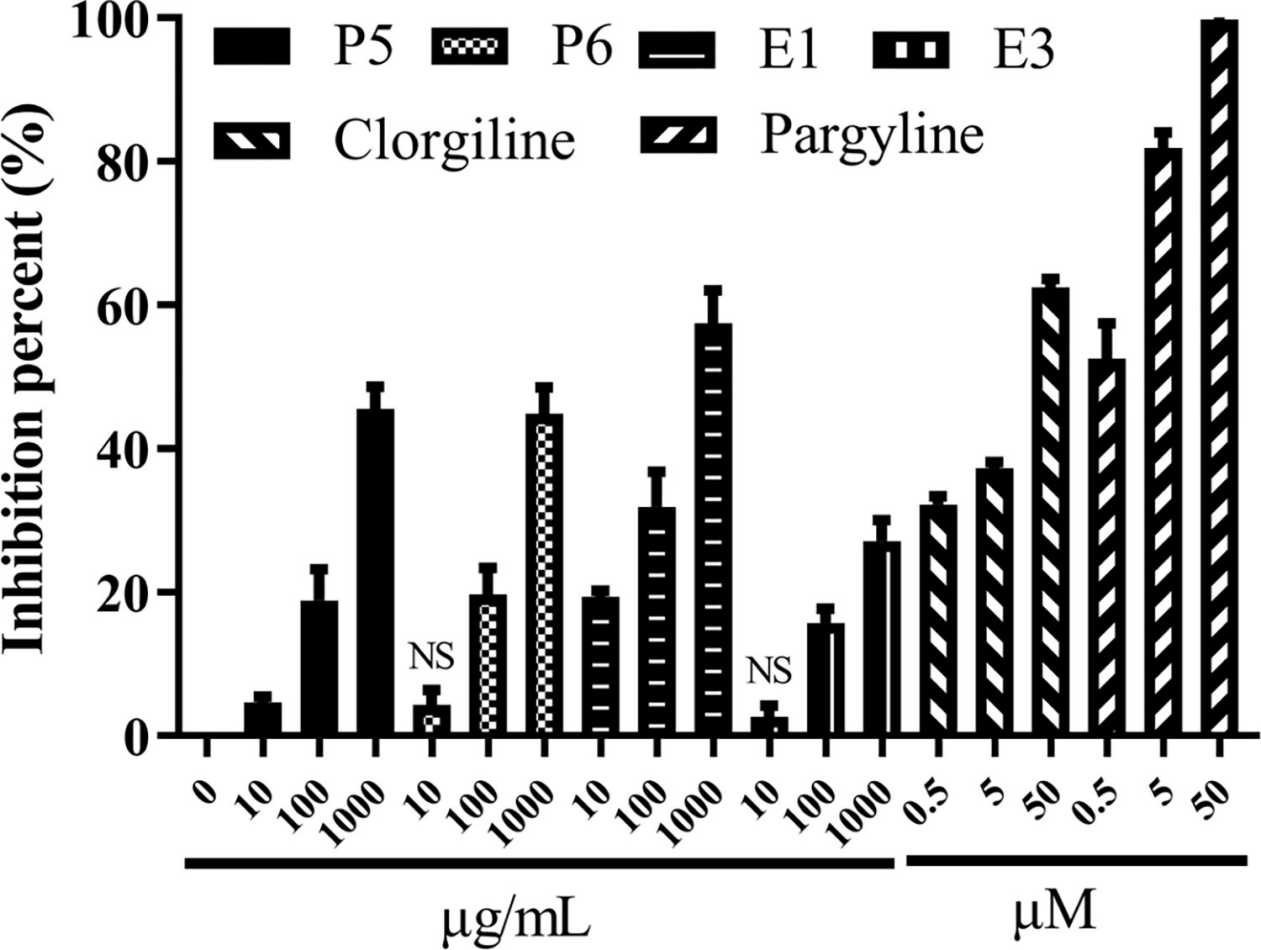
The inhibition percentages of active sub-fractions on MAOs. Data were expressed as mean ± S.E.M. obtained from three independent experiments (n = 3). NS represents the mean of group showed no significant difference with the mean of control group.

The results depicted in Fig 4 showed, MAO-A selective inhibitor—clorgiline could inhibit the MAOs by about 60% at 50 μM, while MAO-B selective inhibitor–pargyline could inhibit the MAOs by close to 100% at 50 μM. Since the enzyme substrate was tyramine, which could be common enzyme substrate for both MAO-A and MAO-B, the enzyme activity of the MAOs we used in this study was considered to be contributed mainly by MAO-B [45].

Except fraction E3, other three active sub-fractions presented more than 40% inhibition percent on MAOs at their highest concentration (1000 μg/mL). The observed inhibition results were regarded as mild.

### SCT sub-fractions inhibit NMDAR

**Fig 5:**
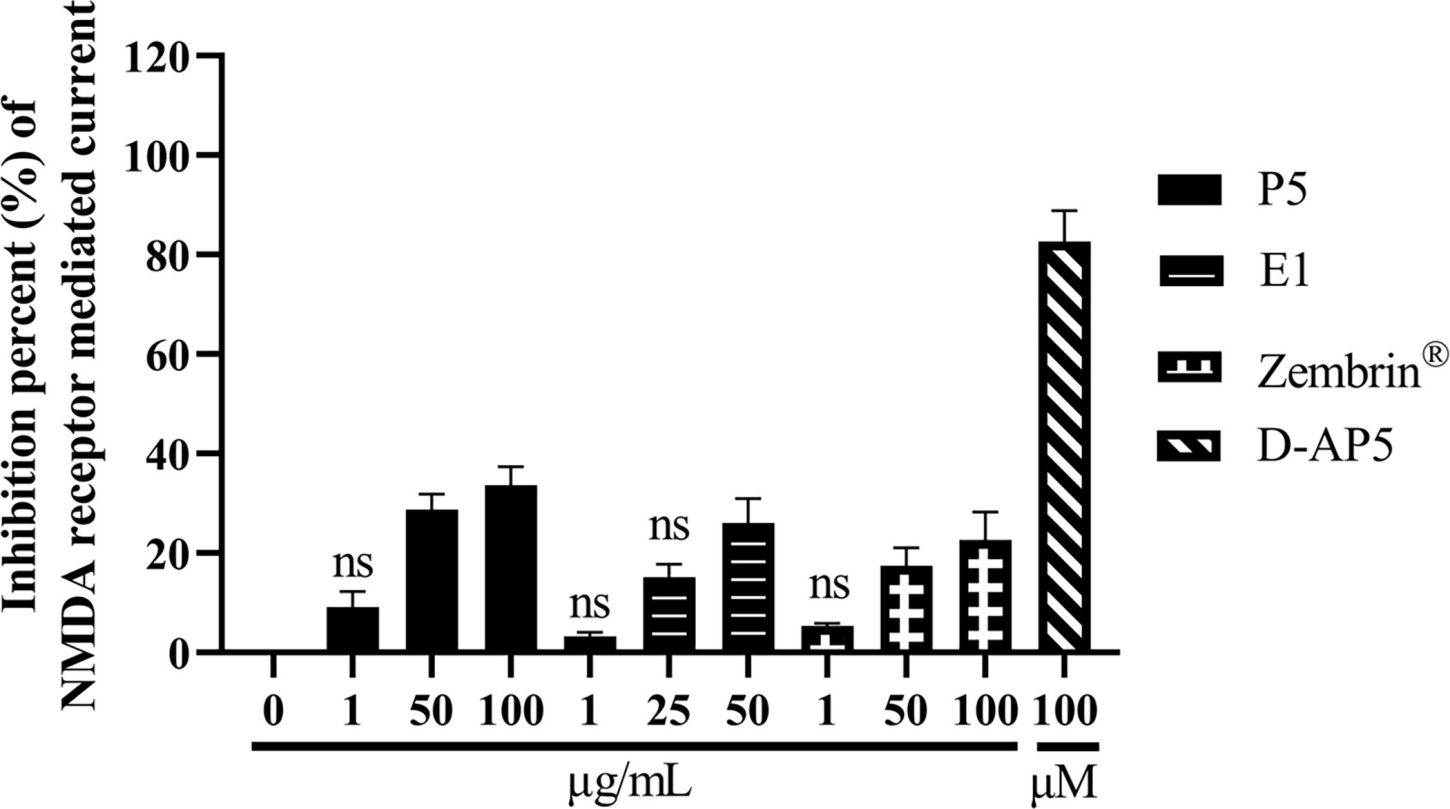
The inhibition percentages of active sub-fractions on NMDAR-mediated current. Data were expressed as mean ± S.E.M. D-AP5 group: n = 4, other groups: n = 3. NS represents the mean of group has no significant different with the mean of control group.

Compared to Zembrin^®^, the more potent neuroprotective P5 and E1 fractions (compared with P6 and E3 in our previous study [20]) showed potent inhibiting effect on NMDAR-mediated current (Fig 5). However, this effect is not significant enough to be considered as main mechanism that elicits antidepressant action of SCT.

### Common targets of constituents and neurodegenerative diseases

**Fig 6:**
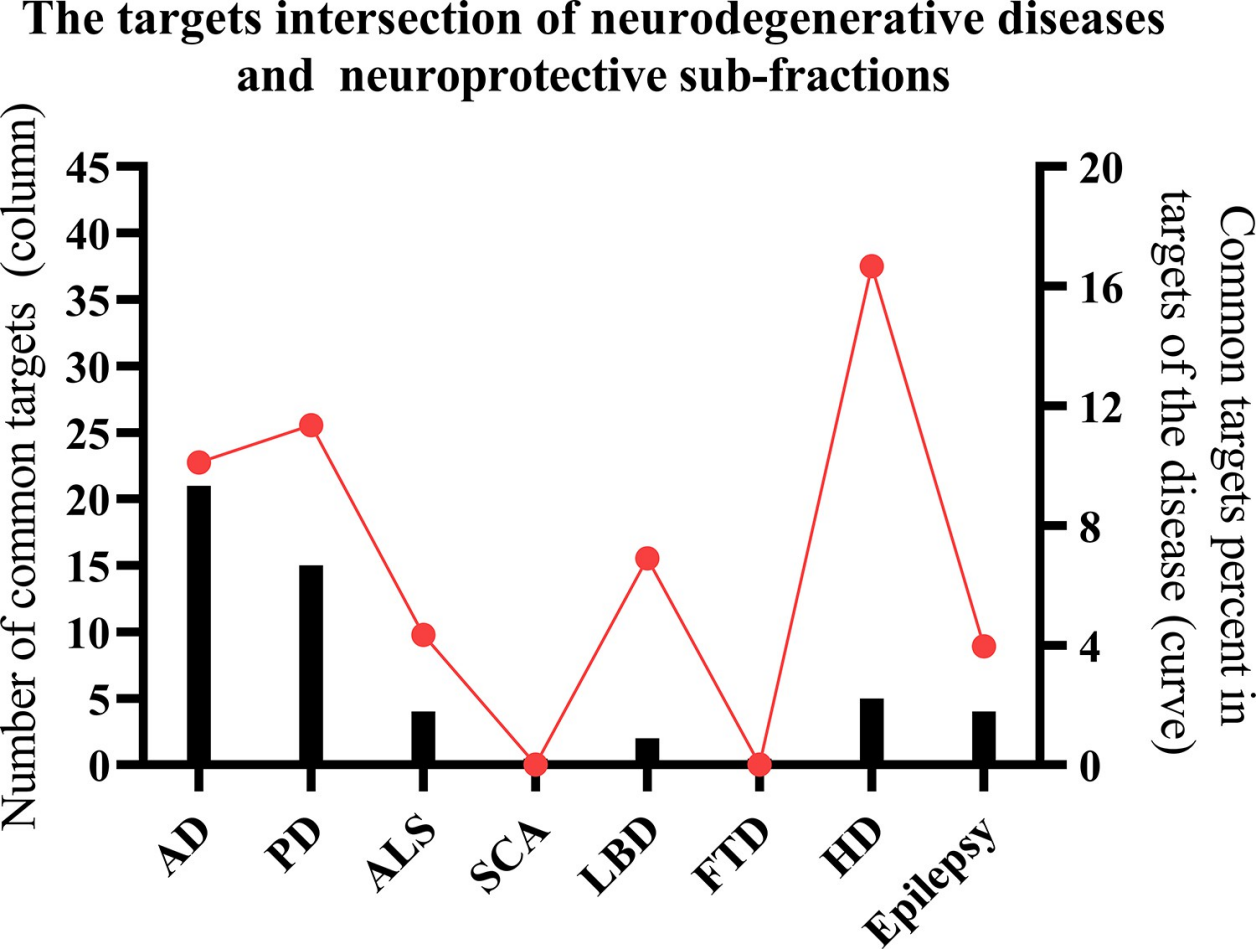
The intersection of targets from constituents and diseases. AD: Alzheimer‘s Disease; PD: Parkinson’s Disease; ALS: Amyotrophic Lateral Sclerosis; SCA: Spinocerebellar Ataxia; LBD: Lewy Body Dementia; FTD: Frontotemporal Dementia; HD: Huntington’s Disease.

As indicated in the previous study, the neuroprotective sub-fractions and underlying neuroprotective constituents (structures were shown in Fig 1) in SCT [20]. Using Polypharmacology Browser 2, the predictive targets of the constituents from neuroprotective sub-fractions were compared with the targets of neurodegenerative diseases collected from GeneCards and DisGeNET databases. The results of their intersections were showed as Fig 6. Although the percent of overlapping targets in targets of HD was the maximum value (16.67%) among these neurodegenerative diseases, there were only 5 overlapping targets from the intersection. Therefore, AD or PD was selected as adaptable disease because of the larger number and percentage of common targets (Table 1) than other disease conditions.

**Table 1:** Overlapping targets of constituents and AD/PD.

Gene	Common name
ESR2	Estrogen receptor beta
MAOB	Monoamine oxidase type B
HTR6	5-hydroxytryptamine receptor 6
CYP2D6	Cytochrome P450 2D6
ACHE	Acetylcholinesterase
SLC6A4	Sodium-dependent serotonin transporter
SLC6A3	Sodium-dependent dopamine transporter
BACE1	Beta-secretase 1
HTR2A	5-hydroxytryptamine receptor 2A
CNR2	Cannabinoid receptor 2
BCHE	Cholinesterase
ALOX5	Polyunsaturated fatty acid 5-lipoxygenase
APP	Amyloid-beta precursor protein
TSPO	Translocator protein
GSK3B	Glycogen synthase kinase-3 beta
PTGS2	Prostaglandin G/H synthase 2
ADAM17	Disintegrin and metalloproteinase domain-containing protein 17
BACE2	Beta-secretase 2
GRIN2B	N-methyl D-aspartate receptor subtype 2B
CTSD	Cathepsin D
TNF	Tumor necrosis factor
HTR1A	5-hydroxytryptamine receptor 1A
DRD3	Dopamine D3 receptor
DRD2	Dopamine D2 receptor
DRD1	Dopamine D1 receptor
ADORA2A	Adenosine receptor A2a
HTT	Huntingtin

### GO and KEGG pathway enrichment analysis

**Fig 7:**
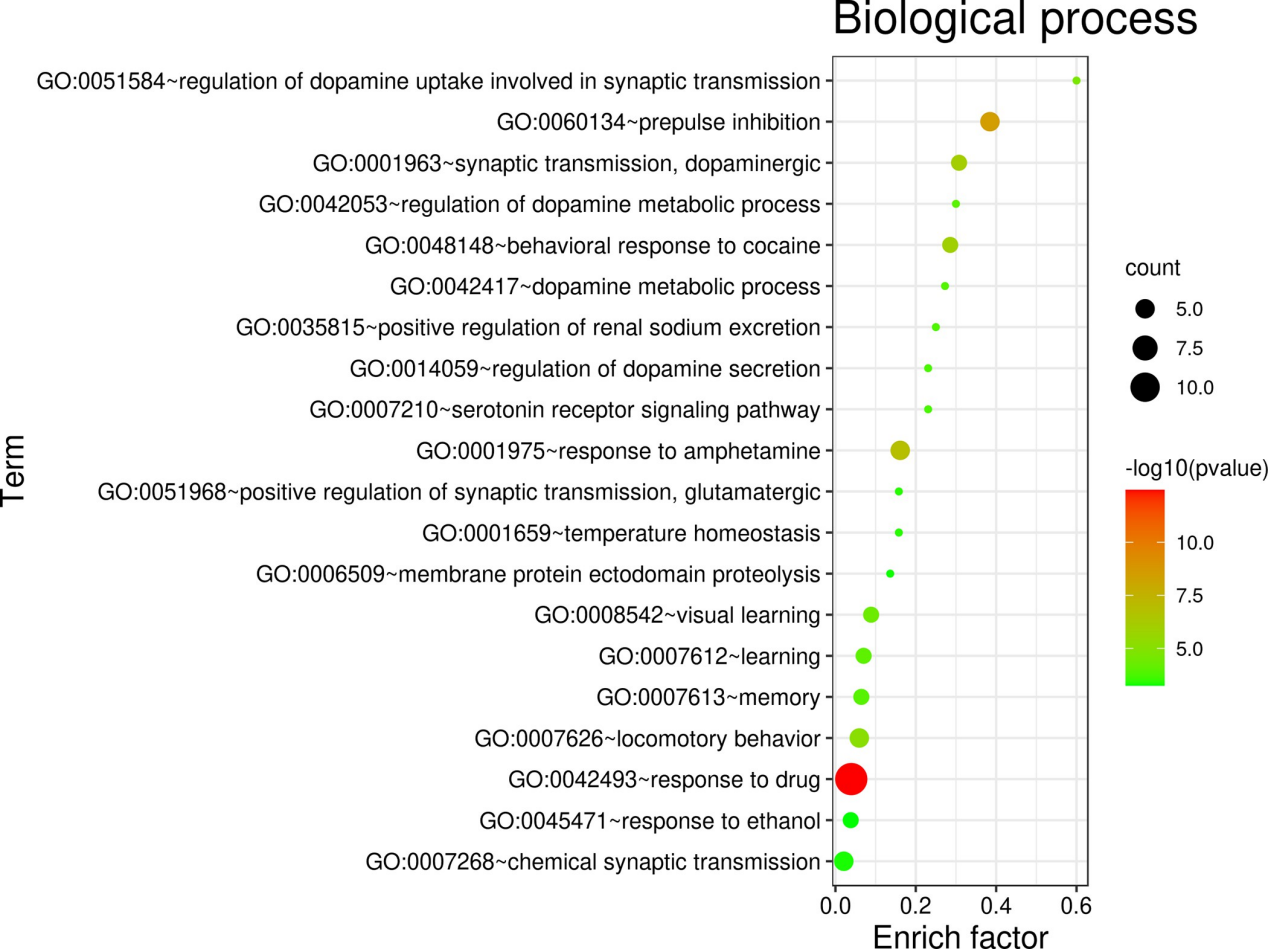
Enrichment analyses for constituents-AD/PD common targets: Biological process.

**Fig 8:**
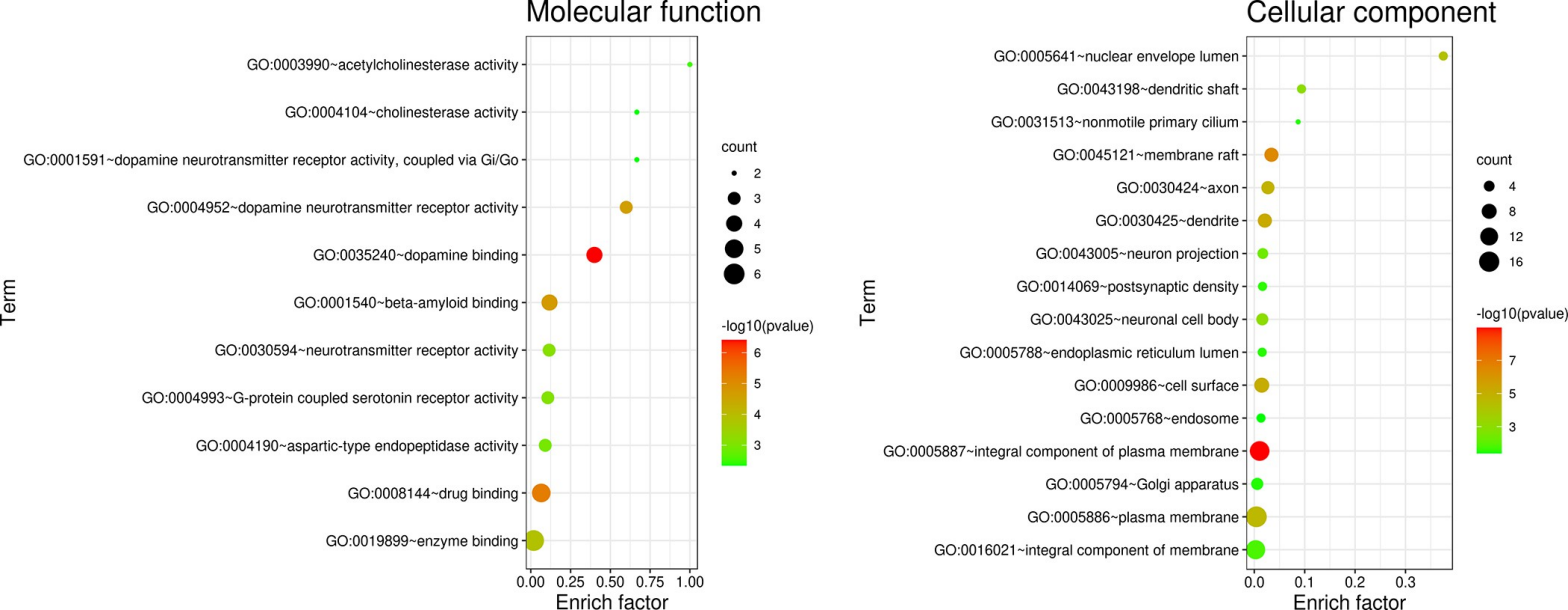
Enrichment analyses for constituents-AD/PD common targets: Molecular function and cellular component.

**Fig 9:**
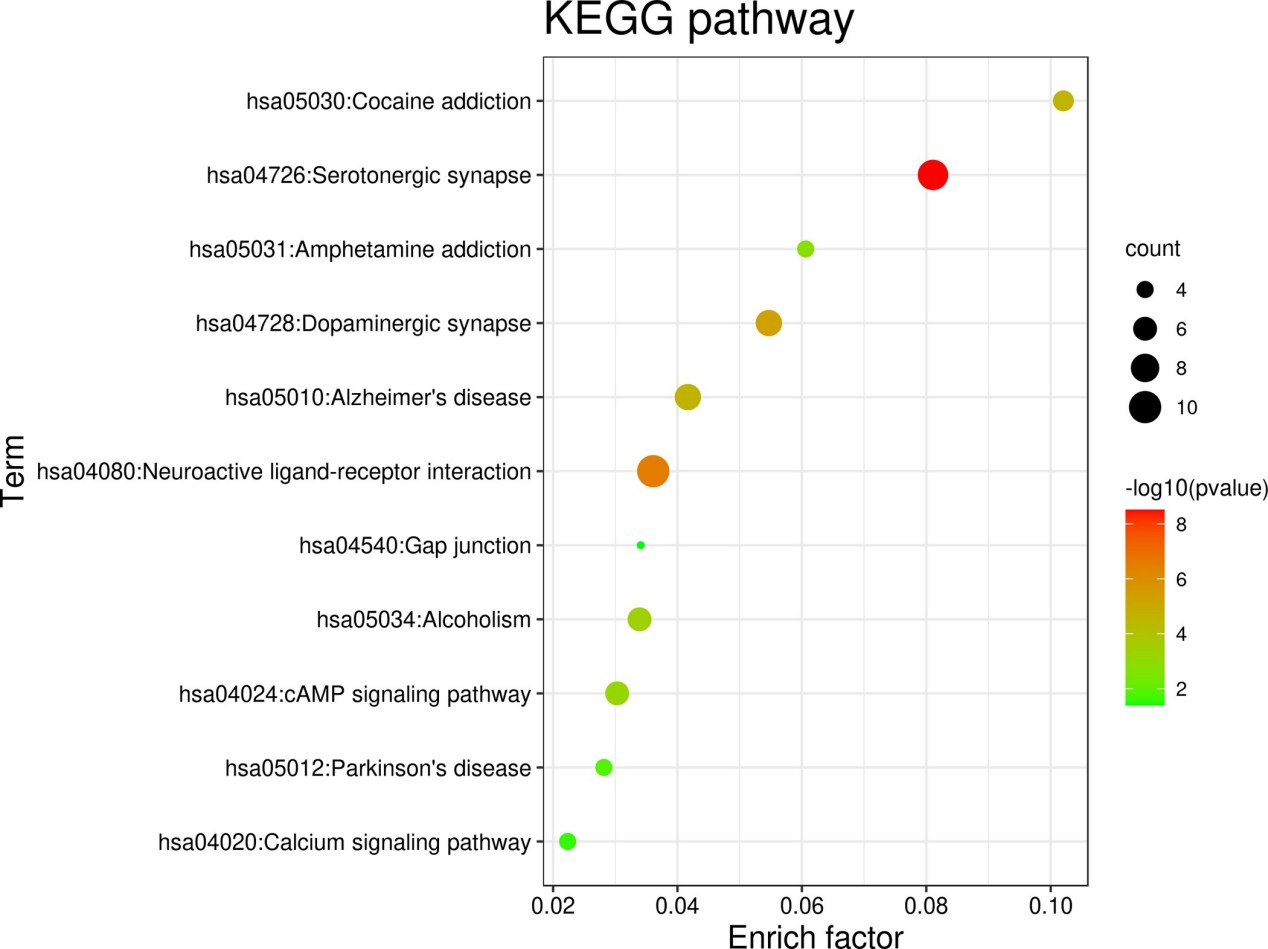
Enrichment analyses for constituents-AD/PD common targets: KEGG pathway.

The overlapping targets of constituents and AD/PD could enrich in more than 20 terms of biological processes (the terms of which P value < 0.001 were showed as Fig 7), which mainly involved response to drug (GO:0042493), chemical synaptic transmission (GO:0007268), locomotory behavior (GO:0007626), memory (GO:0007613), learning (GO:0007612、GO:0008542), response to amphetamine (GO:0001975), behavioral response to cocaine (GO:0048148), dopaminergic synaptic transmission (GO:0001963), prepulse inhibition (GO:0060134), etc. These biological processes indicate that the extracts of SCT could exert neurological activities that are helpful to treat cognition deficit and behavioral disorders. The analysis of cellular functions (Fig 8) showed that these targets mainly included dopamine binding (GO:0035240), dopamine neurotransmitter receptor activity (GO:0004952), beta-amyloid binding (GO:0001540), drug binding (GO:0008144), enzyme binding (GO:0019899), etc. Moreover, these overlapping targets are mainly integral component of plasma membrane (GO:0005887), locate on plasma membrane (GO:0016021) and cell surface (GO:0009986), distribute on dendrite (GO:0030425) and axon (GO:0030424) (Fig 8). KEGG pathway analysis of these targets suggested that they play a role in neuroactive ligand-receptor interaction (hsa04080), serotonergic synapse (hsa04726), dopaminergic synapse (hsa04728), Alzheimer’s disease (hsa05010), alcoholism (hsa05034), cAMP signaling pathway (hsa04024), Parkinson’s disease (hsa05012), calcium signaling pathway (hsa04020), amphetamine addiction (hsa05031) (Fig 9).

### Constituents–targets–disease network diagram

**Fig 10:**
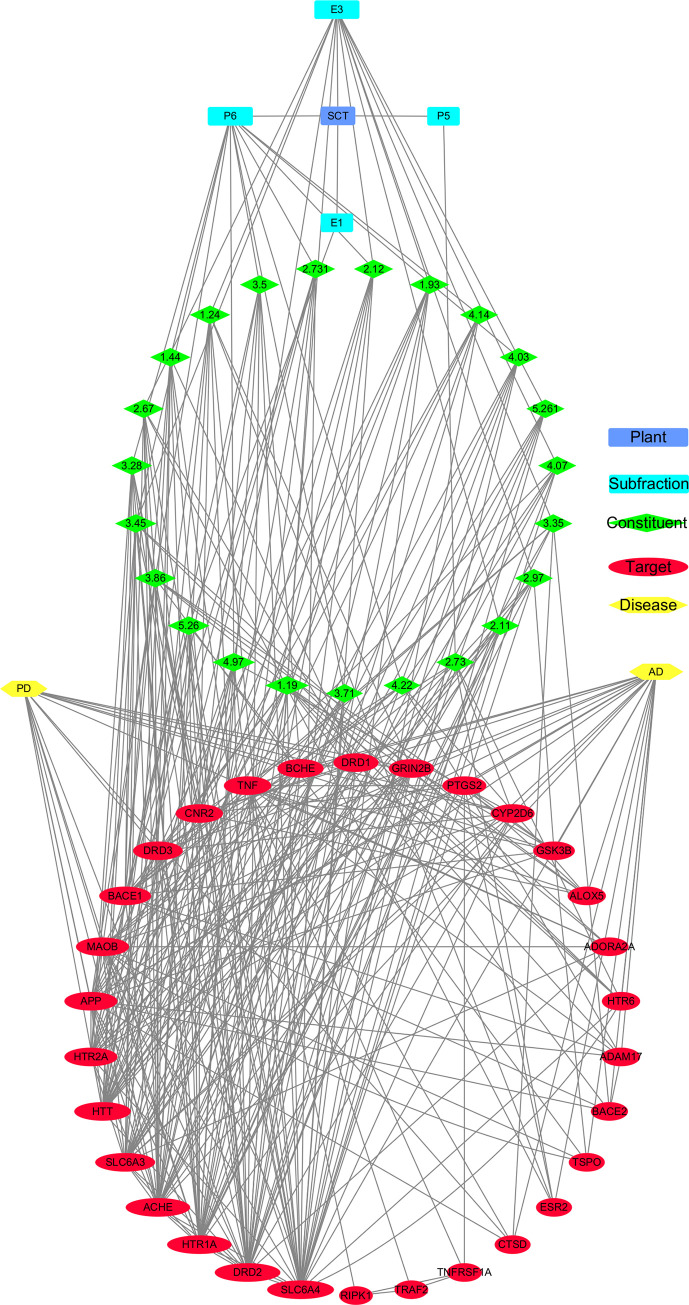
SCT-sub-fraction-constituents-targets-disease network diagram.

The interactions of the overlapping targets, constituents and their possible targets and targets of AD or PD were fed into cytoscape 3.8.0 software to obtain a constituents-targets-disease network diagram (Fig 10). In this network diagram, there were 59 nodes and 345 edges, including 23 constituents, 30 targets, 2 diseases, 4 sub-fractions and 1 plant. The result of network analysis (Table 2) showed that degrees of the targets, of which gene names are SLC6A4, DRD2, ACHE, HTR1A, SLC6A3, HTT, APP, HTR2A, MAOB, BACE1, DRD3, TNF, CNR2, BCHE, DRD1 and GRIN2B, are more than 13 with betweenness centralities more than 0.005 and closeness centralities more than 0.5. The degrees of all constituents in this diagram are equal or greater than 6.

**Table 2:** The results of topological analysis for the network.

Node Name	Degree	Betweenness Centrality	Closeness Centrality
SLC6A4	32	0.06260	0.62766
DRD2	31	0.05874	0.64130
ACHE	30	0.10778	0.65556
HTR1A	30	0.07519	0.64130
SLC6A3	26	0.03614	0.59596
HTT	23	0.03893	0.57282
APP	20	0.04156	0.57843
HTR2A	20	0.02568	0.56731
MAOB	20	0.02614	0.55140
BACE1	19	0.05334	0.57282
DRD3	17	0.01538	0.55660
TNF	15	0.08981	0.52679
CNR2	15	0.02514	0.55140
BCHE	14	0.01292	0.53636
DRD1	13	0.00678	0.52212
GRIN2B	13	0.00617	0.52212
PTGS2	12	0.02749	0.51754
CYP2D6	12	0.00583	0.53153
GSK3B	10	0.01317	0.47967
ADAM17	7	0.00234	0.45385
ADORA2A	7	0.00621	0.50000
ALOX5	7	0.00475	0.45385
HTR6	7	0.00442	0.49167
BACE2	6	0.00107	0.43704
CTSD	5	0.00113	0.42143
ESR2	5	0.00722	0.42143
TSPO	5	0.00034	0.41259
TNFRSF1A	4	0.00171	0.36646
RIPK1	3	0	0.35119
TRAF2	3	0	0.35119
1.19	12	0.02202	0.51304
4.97	11	0.00727	0.50000
3.45	10	0.00977	0.50000
3.86	10	0.01547	0.50000
5.26	10	0.03077	0.50427
1.44	9	0.00425	0.49580
3.28	9	0.00307	0.50000
1.24	9	0.00949	0.49580
2.67	9	0.00869	0.48361
2.12	8	0.00342	0.48361
2.731	8	0.00244	0.49167
3.50	8	0.00250	0.47967
1.93	8	0.00212	0.45385
2.73	7	0.02801	0.45385
4.03	7	0.00167	0.47581
4.14	7	0.00193	0.47581
2.11	7	0.00152	0.45038
2.97	7	0.00471	0.47967
3.35	7	0.01321	0.50427
4.07	7	0.00184	0.48361
5.261	7	0.00153	0.47200
3.71	6	0.00130	0.46825
4.22	6	0.00612	0.45385

### Key targets in the possible mechanisms of SCT in the treatment of AD or PD

**Fig 11:**
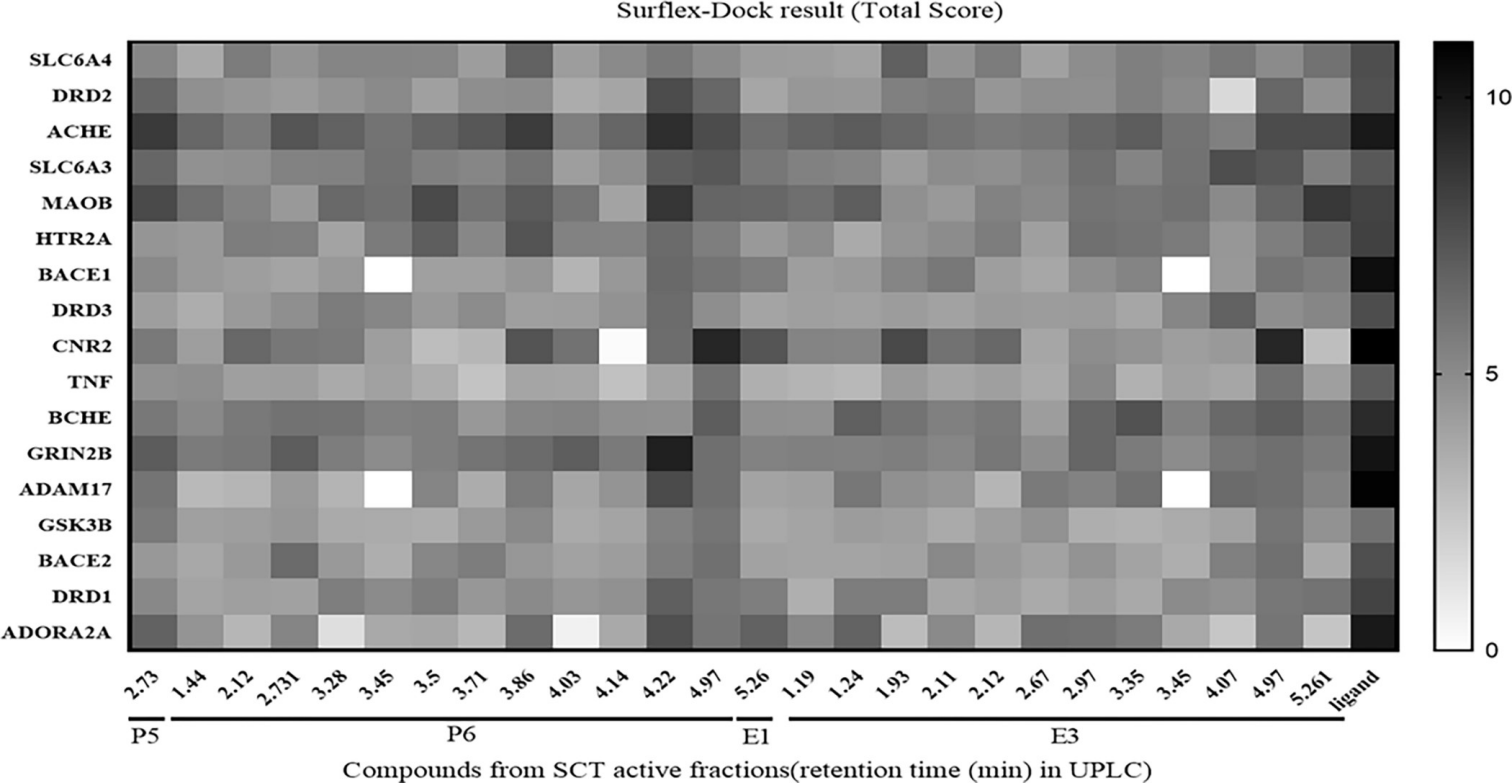
Surflex-dock results of SCT constituents with key targets in total score.

Targets with a greater degree value (more than 13) or enriched in AD or PD KEGG pathway were selected to be docked with constituents from neuroprotective sub-fractions by Surflex-Dock (Total Score results showed as Fig 11).

**Fig 12:**
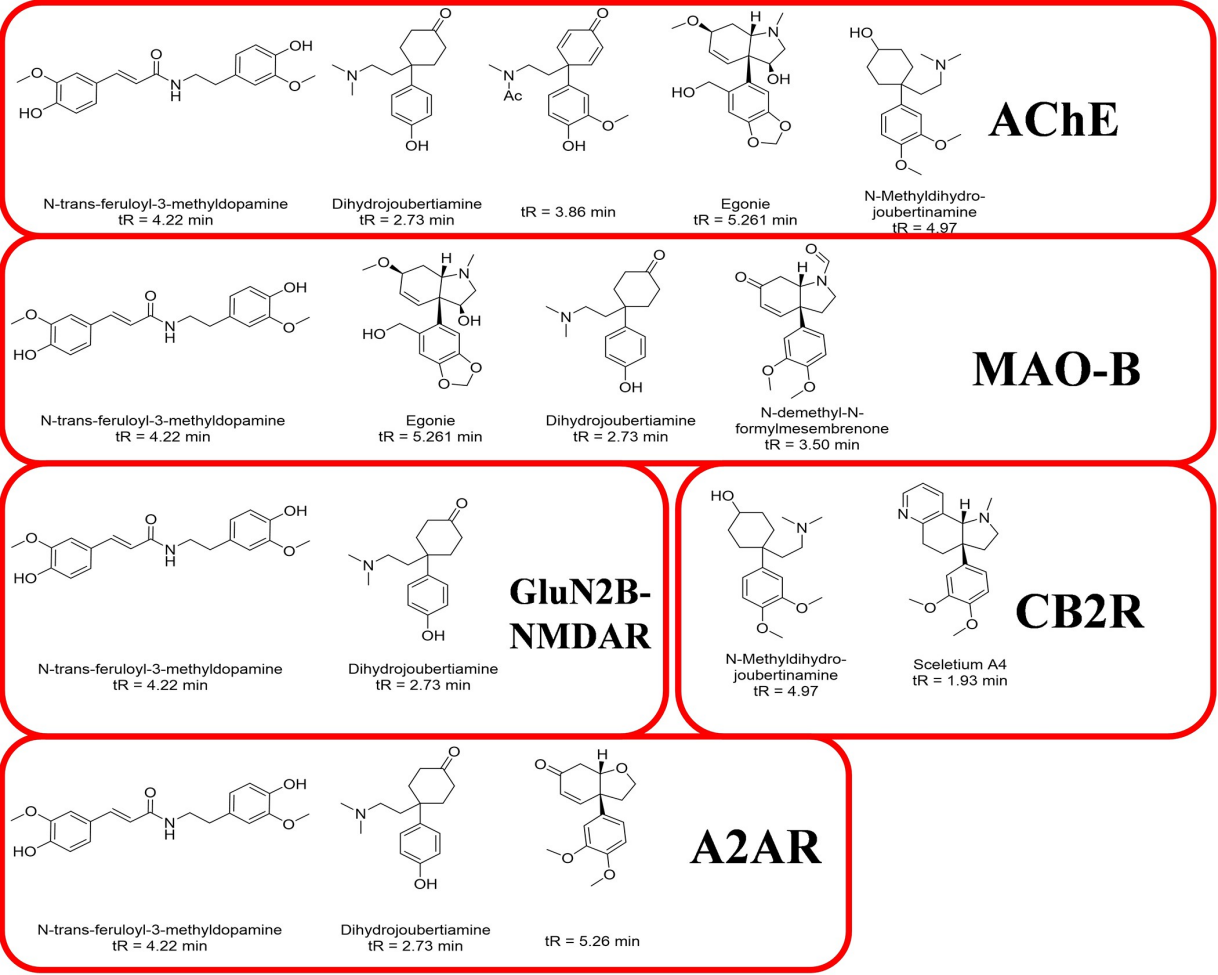
Constituents from SCT with their possible targets predicted by total score in surflex-dock.

The Total Score results indicated that many vital targets involved in AD or PD, for example AChE (ACHE), MAO-B (MAOB), GluN2B-NMDAR (GRIN2B), adenosine A2A receptor (A2AR, ADORA2A) and cannabinoid receptor 2 (CB2R, CNR2), have potent predicted binding activity with several SCT constituents. Moreover, SCT constituents as Fig 12 showed have higher Total Score with corresponding targets, which indicated that they are more possible to affect the targets to exert neuroprotective efficacy for the treatment of AD or PD.

## Discussion

The outcomes of this study demonstrated the efficacies of SCT neuroprotective sub-fractions in scavenging DPPH radical, inhibiting AChE, MAOs and NMDAR by experiments performed using *in vitro* models.

The clearance percent of four sub-fractions could reach more than 40% at 500 μg/mL. In contrast to the radical scavenging efficacy of SCT extract in the previous study, E3 could present comparative performance on scavenging DPPH radical [46], which indicated the constituents with antioxidant effect of SCT was enriched in the ethyl acetate sub-fraction. Antioxidative effect is a known mechanism of certain compounds eliciting neuroprotective action [7, 47–49]. The results further suggest that SCT has potential to treat neurodegenerative disorders through its antioxidative effect.

The study also showed moderate inhibiting effect of SCT neuroprotective sub-fractions on AChE, which was more potent than the effect of SCT extract in previous study based on comparing their test concentrations [50]. The reduction of acetylcholine level in AD patient may cause cognitive and memory impairments [51]. Hence, AChE may accelerate the progression of AD though promoting the fibration of β-amyloid [52]. Scopolamine, a muscarinic receptor antagonist, produces a blocking of the activity of the muscarinic acetylcholine receptor, and the concomitant appearance of transient cognitive amnesia and electrophysiological changes, which resemble those observed in AD [53, 54]. There are certain AChE inhibitors approved for AD, for example, donepezil and galanthamine. In fact, some studies had described the neuroprotective effect of AChE inhibitor [22, 23]. Hence, inhibiting AChE appears to be an underlying mechanism of the neuroprotective action of SCT.

The results of MAOs inhibiting assay showed, except E3, other three active sub-fractions (P5, P6 and E1) all presented more than 40% inhibition percent on MAOs at 1000 μg/mL, which is still more potent than the inhibiting effect of SCT extract on MAO-A by comparing their concentrations [50]. Since MAO-A selective inhibitor (clorgyline) could not inhibit the crude MAOs close to 100% at 50 μM, while MAO-B selective inhibitor (pargyline) could inhibit it close to 100% at at 50 μM. This result indicated that enzyme activity of the crude MAOs used in this study mainly contributed by MAO-B [39, 45]. Excess MAOs catalyze oxidation of amino substance causing the generation of oxidative stress [55, 56]. Moreover, a MAO-B inhibitor–selegiline approved for PD was reported to suppress excess GABA produced from astrocytes and restores the impaired spike probability, synaptic plasticity, and learning and memory in the mice [24]. However, some clinical trials showed that the cognitive function of the placebo group had no significant difference compared to the group treated with selegiline for a long term therapy [25, 26]. Instead of irreversible inhibitor like selegiline, a reversible MAO-B inhibitor (KDS2010) does not induce compensatory mechanisms in a long term therapy, which further attenuated increased astrocytic GABA levels and astrogliosis, enhanced synaptic transmission, rescued learning and memory impairments in APP/PS1 mice [27]. Thereby, MAO-B is considered as a key target of SCT in the treatment of AD or PD.

Furthermore, P5 and E1 fractions SCT presented a non-significant inhibition of NMDAR-mediated current in hippocampal neurons of Sprague-Dawley neonatal rats, which was more potent than the effect of Zembrin^®^ on NMDAR-mediated current and consistent with previous results [57]. NMDAR, an ionotropic glutamate receptor, which constitute a calcium-permeable component of fast excitatory neurotransmission, have been verified to participate neuro-physiologically in many cell signaling pathways resulting in several neurological diseases. An NMDAR inhibitor–esketamine was approved for depressive disorder ought to his rapid antidepressant action. The previous studies showed the potential of SCT on treating depressive disorder [2, 4, 28–33]. However, the results of this study indicated that the influence on NMDAR of these two fractions may be a subsequent effect resulted from affecting other targets but not NMDAR. Thus leading to a possibility that the anti-depressive action of SCT extract can be due to inhibition efficacy of mesembrine and mesembrenone on phosphodiesterase-4 and serotonin transporter [58].

Therefore, results of the *in vitro* experiments indicated that there are neuroprotective constituents in SCT could protect neurons to treat neurodegenerative disorders by scavenging radicals, inhibiting AChE, MAOs and NMDAR. Different sub-fractions represented different degrees of influence on AChE, MAOs and NMDAR.

Moreover, the neuroprotective sub-fractions of SCT used to assess the potential use to treat AD or PD, was further supported by network pharmacology related methods applied in this study, which was also supported by the observed influence of SCT extract on cognition [5, 6]. Among several neurodegenerative disorders, the targets of AD or PD from database have most overlapping numbers with the targets predicted by Polypharmacology Browser 2. It is understood that the overlapping targets could be involved in memory, learning and behavior related biological process and enrich in AD and PD corresponding KEGG pathway. The network analysis and Surflex-Dock results have indicated that some key targets, AChE, MAO-B, GluN2B-NMDAR, A2AR and CB2R, can be influenced by SCT in the probable treatment of AD or PD, and other constituents SCT or similar moieties of close chemical structures, such as egonie, sceletium A4, dihydrojoubertiamine, N-trans-feruloyl-3-methyldopamine, N-methyldihydrojoubertinamine and so on, should be concerned to have potential in affecting on corresponding targets (Fig 12).

In this study, the primary purpose was to explore possible targets of the neuroprotective SCT on neurodegenerative disorders by network pharmacology. According to the identified constituents from SCT in our previous study, the results of network pharmacology studies indicated some potential targets (AChE、MAOs and NMDAR) for SCT. Therefore, the neuroprotective SCT sub-factions were further tested in vitro for their efficacy on the potential targets. Encouragingly, the results of the fraction-targets in vitro experiments actually supported the network pharmacology results in this study. However, different sub-factions contained different natural products, the content of natural products were also various, which resulted in the different effects of different sub-factions to these potential target in this study. Certainly, in the next stage, the further studies would carry out to explain the bioactivity mechanism of different sub-fractions on their specific targets.

## Conclusion

SCT neuroprotective sub-fractions have moderate potency of scavenging radicals, inhibiting AChE, MAOs and NMDAR, which are the possible mechanisms of its neuroprotective effect. The identified and other related constituents in SCT may have affects on biological systems to alter AChE, MAO-B, GluN2B-NMDAR, A2AR and CB2R, to exert their therapeutic potential in the probable treatment of AD or PD.

## Abbreviations

SCT: Sceletium tortuosum
MPP^+^: 1-methyl-4-phenylpyridinium
AChE: Acetylcholinesterase
MAO: monoamine oxidase
NMDAR: N-methyl-D-aspartic acid receptor
AD: Alzheimer’s disease
PD: Parkinson’s disease
DPPH: 1,1-diphenyl-2-picrylhydrazyl
A2AR: adenosine A2A receptor
CB2R: cannabinoid receptor 2
KEGG: Kyoto Encyclopedia of Genes and Genomes
